# Proteomic Identification of DNA-PK Involvement within the RET Signaling Pathway

**DOI:** 10.1371/journal.pone.0127943

**Published:** 2015-06-11

**Authors:** Lyle J. Burdine, Marie Schluterman Burdine, Linley Moreland, Brad Fogel, Lisa M. Orr, Jennifer James, Richard H. Turnage, Alan J. Tackett

**Affiliations:** 1 Department of Surgery, University of Arkansas for Medical Sciences, Little Rock, AR 72205, United States of America; 2 Department of Biochemistry and Molecular Biology, University of Arkansas for Medical Sciences, Little Rock, AR 72205, United States of America; 3 Department of Pathology, University of Arkansas for Medical Sciences, Little Rock, AR 72205, United States of America; Hungarian Academy of Sciences, HUNGARY

## Abstract

Constitutive activation of the Rearranged during Transfection (RET) proto-oncogene leads to the development of MEN2A medullary thyroid cancer (MTC). The relatively clear genotype/phenotype relationship seen with RET mutations and the development of MEN2A is unusual in the fact that a single gene activity can drive the progression towards metastatic disease. Despite knowing the oncogene responsible for MEN2A, MTC, like most tumors of neural crest origin, remains largely resistant to chemotherapy. Constitutive activation of RET in a SK-N-MC cell line model reduces cell sensitivity to chemotherapy. In an attempt to identify components of the machinery responsible for the observed RET induced chemoresistance, we performed a proteomic screen of histones and associated proteins in cells with a constitutively active RET signaling pathway. The proteomic approach identified DNA-PKcs, a DNA damage response protein, as a target of the RET signaling pathway. Active DNA-PKcs, which is phosphorylated at site serine 2056 and localized to chromatin, was elevated within our model. Treatment with the RET inhibitor RPI-1 significantly reduced s2056 phosphorylation in RET cells as well as in a human medullary thyroid cancer cell line. Additionally, inhibition of DNA-PKcs activity diminished the chemoresistance observed in both cell lines. Importantly, we show that activated DNA-PKcs is elevated in medullary thyroid tumor samples and that expression correlates with expression of RET in thyroid tumors. These results highlight one mechanism by which RET signaling likely primes cells for rapid response to DNA damage and suggests DNA-PKcs as an additional target in MTC.

## Introduction

Medullary thyroid carcinoma (MTC) arises from neural crest-derived parafollicular C-cells located in the basal layer of thyroid follicles and accounts for 5–10% of thyroid cancers. 20–30% of MTC are a hereditary form known as multiple endocrine neoplasia type 2A or B (MEN2A, 2B) and familial medullary carcinoma (FMTC) [1, 2]. Total thyroidectomy and lymph node dissection is the curative treatment for MTC. However, advanced metastatic MTC is inoperable with a 5-yr. survival rate less than 28% (American Cancer Society, 2010). Additional investigation into the cellular mechanisms driving MTC chemoresistance is warranted.

98% of MEN2A cases are caused by germline mutations in the RET (REarranged during Transfection) proto-oncogene that encodes for a receptor tyrosine kinase, which exists in one of two alternatively spliced variants, RET 9 or RET 51 [2,3]. Although the two variants differ by the addition of either 9 or 51 residues at the C-terminal, they display similar downstream signaling effects. Upon dimerization with its ligand, glial cell line–derived neurotrophic factor (GDNF) family ligand (GFL), RET is activated through autophosphorylation of intracellular tyrosine residues. Activation results in stimulation of signaling pathways involved in cell motility, proliferation, differentiation and survival via the mitogen activated protein kinase (RAS/MAPK) and phosphoinositide 3-kinase (PI3K)/AKT pathways [4]. RET mutations found in MEN2A are dominant gain of function mutations that allow for RET to self-dimerize and be constitutively active [2]. Activation leads to tumorigenesis by upregulating cell division and proliferative processes. The RET signaling program has been studied extensively and multiple clinical trials have been carried out using receptor tyrosine kinase inhibitors or RET small molecule inhibitors to treat MEN2A. Although there is a clear genotype/phenotype relationship seen with RET mutations and the development of MEN2A, these drugs only resulted in marginal response and advanced metastatic MEN2A remained largely resistant [5, 6]. We are interested in investigating the mechanism by which those cells executing a RET oncogenic program increase their capacitance to absorb genotoxic stress.

Evidence suggests that changes to chromatin structure are the earliest events to occur in the transformation of a normal cell [7]. We hypothesized that the constitutive activation of RET drives the formation of MEN2A by altering chromatin machinery (e.g., DNA damage repair) to favor the generation of a highly drug resistant cell population. This work set out to elucidate responsible mechanisms.

## Materials and Methods

### Cell culture

SK-N-MC cells stably expressing either a RET expression construct containing the extracellular and transmembrane domains of the EGF receptor linked to either the RET9 isoform, RET51 isoform intracellular domains or an empty vector were kindly provided by Dr. Michael Skinner (UT Southwestern Medical Center, Dallas, TX). Unmodified SK-N-MC cells are an established cell line obtainable from ATCC (Manassas, VA). Cells were maintained at 95% air and 5% CO_2_ at 37°C and grown in MEMS medium (Life Technologies, Grand Island, NY) supplemented with 10% FBS (Atlanta Biologicals, Flowery Branch, GA), pen/strep, and 400 μg of G418 (Hyclone, Logan, UT) for continued selection. Cells were routinely checked for RET expression by western blot analysis. TT cells (CLR-1803) were obtained from ATCC and maintained in F-12K medium with 10% FBS and pen/strep.

### Nuclei isolation

TT cells were grown in 6-well plates for nuclei isolation. Cells were washed twice with ice cold PBS, scraped and centrifuged at 3000 rpm for ten minutes. PBS was removed and pellets were resuspended in hypotonic lysis buffer (10 mM Tris-HCL pH 7.9, 1.5 mM MgCl2, 5 mM KCl, protease inhibitors and phosphatase inhibitors (Roche, Indianapolis, IN)) and incubated on ice for ten minutes. Lysates were centrifuged for five minutes at 6000 rpm at 4°C. Supernatant containing the cytoplasmic fraction was removed and the pellets were resuspended in nuclear extraction buffer (50 mM Tris-HCL pH 7.9, 0.5 M NaCl, 2mM EDTA, 10% sucrose, 10% glucose, protease inhibitors and phosphatase inhibitors) and incubated on ice for 30 minutes with vortexing every ten minutes. Lysates were centrifuged for 30 minutes at 14,000 rpm and the supernatant containing nuclei were stored at -20°C until they were used for western blot.

### Western blot

Cells were plated in 6-well culture dishes (6X10^5^ cells/well) and allowed to adhere overnight. Protein lysates were obtained by rinsing cells once with PBS and lysing cells with a Triton X-100 lysis buffer (40 mM Tris pH 7.4, 120 mM NaCl, 0.5% Triton X-100, 0.3% SDS, protease inhibitors and phosphatase inhibitors (Roche, Indianapolis, IN). Lysates were resuspended in 2x BME sample buffer and boiled for five minutes prior to being separated on SDS-PAGE Tris-glycine 4–20% gradient gels (Bio-Rad, Hercules, CA). For analysis of DNA-PKcs, nuclear lysates were separated on NuPage 3–8% Tris-Acetate gels (Bio-Rad). Gels were transferred onto nitrocellulose membranes for 3 hours at 30 V (Tris-Acetate gels were transferred overnight in the cold room at 15 V). Immunoblotting was performed using the following antibodies: RET 9, RET (C-19) sc-167 (Santa Cruz, Dallas, TX), RET 51, RET (C-10) sc-1290 (Santa Cruz), p-RET (Tyr 1062)-R sc-20252-R (Santa Cruz), Akt1 (C73H10) #2938 (Cell Signaling, Danvers, MA), p-Akt (ser473) (193H12) #4058 (Cell Signaling), GAPDH (14C10) #2118S (Cell Signaling), DNA-PKcs ab53701 (Abcam, Cambridge, MA), p-DNA-PKcs (p-S2056) ab18192 (Abcam), Lamin B1 ab16048 (Abcam) and pERK 4370S (Cell Signaling). All HRP-conjugated secondary antibodies were from Jackson Immuno Laboratories and used at 1:25000. For treatment with the RET inhibitor, RPI-1 (R8907, Sigma-Aldrich) at 50 μM (RET cells) and 5 μM (TT cells) was supplemented in the media for 24 hours prior to harvesting cells for western blot analysis.

### Cell Viability Assay

To monitor the sensitivity of RET cells or TT cells to chemotherapy cell viability assays were performed. Empty vector (EV, control) cells along with cells containing RET 9 and RET 51 isoforms were plated in 96-well plates (10K/well) and allowed to adhere overnight. Medium was replaced with medium containing doxorubicin (DOX) (D1515, Sigma Aldrich, St. Louis, MO) at 0 nM, 100 nm, 250 nM and 500nM. TT cells were also plated in 96-well plates (10K/well) and allowed to adhere overnight before DOX was added to fresh medium at 100nM. In assays looking at the effect of DNAPKcs inhibition, cells were treated with NU7441 inhibitor (KU-57788, Selleckchem, Houston, TX) with or without DOX at 1.0 and 2.0 μM (1 μM for TT cells). After 24 hours, plates with RET cells were removed from the incubator and measured for cell viability using the CellTiter-Glo Luminescent Cell Viability Assay kit (# G7572 Promega, Madison, WI) according to the manufacturer’s protocol. Due to the slow growth rate of TT cells, the assays were performed 4 days after drug was applied. Luminescence was measured on a Perkin Elmer Victor 3V 1420 Multilabel plate reader. The assay was performed in triplicate and statistical analysis performed by standard ttests.

### Histone extraction from isolated nuclei

Histones and chromatin binding proteins were isolated from EV, RET 9 and RET 51 cells by H2SO4 extraction. Nuclei were isolated from 1X10^7^ cells of each cell line and washed in 1X PBS then centrifuged for five minutes at 2000 rpm. Cells were resuspended in 1 ml of hyptonic lysis buffer (10 mM Tris-HCL pH 8.0, 1 mM KCl, 1.5 mM MgCl2, 1 mM DTT, 0.4 mM PMSF, protease and phosphatase inhibitors). Cell lysis mix was rotated for 30 minutes at 4°C. Following lysis, samples were centrifuged for ten minutes at 10,000 rpm and the nuclei were resuspended in 200 μl of 0.4N H2SO4. Samples were allowed to mix overnight at 4°C. The next day, samples were centrifuged for ten minutes at 13, 000 rpm at 4°C and extracted histone pellets were resuspended in 66 ul of 100% TCA and incubated on ice for 30 minutes. Histones were centrifuged for ten minutes at 13,000 rpm and washed twice in ice cold 100% acetone. Pellets were allowed to completely air dry before being resuspended in H20. Extraction was performed in biological triplicate for mass spectrometry analysis.

### Mass Spectrometry

Preparation of protein samples and analysis of data was performed as previously reported by our laboratory and detailed in [8–10]. In short, ten μg of histones were separated on a 4–12% Bis-Tris gradient gel (Life Technologies) and stained with Coomassie Blue (manufacturer’s protocol, Bio-Rad) to visualize proteins. Bands were cut out of the gel and gel pieces containing histones and nuclear associated proteins were diced into smaller pieces for in-gel trypsin digest. Gel pieces were destained in 50% methanol, 100 mM ammonium bicarbonate, followed by reduction in 10 mM Tris[2-carboxyethyl] phosphine and alkylation in 50 mM iodoacetamide. Gel slices were then dehydrated in acetonitrile, followed by addition of 100 ng porcine trypsin (Promega) in 100 mM ammonium bicarbonate and incubation at 37°C for ~14 hours. Peptide products were then acidified in 0.1% formic acid. Tryptic peptides were analyzed by nanoflow LC-MS/MS with a Thermo Orbitrap Velos mass spectrometer equipped with a Waters nanoACQUITY LC system. A total of 486 proteins were identified by a Mascot human database search. Mascot results were uploaded into Scaffold 4 (version 4.00.01) for viewing the proteins and peptide information. A false discovery rate of 1% was used as the cut off value. In order to identify proteins significantly altered between our control lines and those overexpressing RET 9 and 51, we used spectral counting normalized by the Normalized Spectral Abundance Factor (NSAF) method described previously by Byrum S. et al. [8]. Following log transformation of NSAF values, standard Ttests were performed to identify significant proteins. A p-value of < 0.05 was the cutoff for significance.

### Immunocytochemistry

EV, RET 9 and RET 51 cells were plated in 8-well chamber slides (Fisher Sci., Pittsburgh, PA) at 5 X 10^4^ and allowed to adhere overnight. The following day medium was removed and cells washed once in PBS. Cells were fixed in acetone for twenty minutes at -20°C. Cells were washed once in PBS and blocked in 5% FBS/PBS for one hour at room temperature. Following blocking, primary antibody was added to cells (p-DNA-PKcs (p-S2056) ab18192 (Abcam)) at 1:50 dilution and incubated overnight at 4°C. Primary antibody was removed and cells were washed gently with PBSt 3 X 5 minutes at room temperature. Secondary antibody (Cy3-AffiniPure donkey anti-rabbit antibody, Jackson ImmunoResearch, 711-165-152) at a 1:1000 dilution was applied for one hour at room temperature. Cells were washed 3 X 5 minutes in PBSt and counterstained with DAPI (Prolong Gold antifade, P36931, Life Technologies) then coverslipped. Images were taken with a Zeiss AxioImager Z1 microscope and an attached Zeiss AxioCam MRc5 camera fitted with a Cy3 filter. Ten 20X images were taken of each condition. A blind observer recorded the total number of nuclei along with the number of positive nuclei. Data is displayed as % positive nuclei. Standard Ttests were performed for statistical analysis.

### Immunohistochemistry

Parafin-embedded formalin-fixed human thyroid tissue microarrays were purchased from US BioMax Inc. (Rockville, MD). Staining was performed by the UAMS Pathology Core following standard staining methods. Antibodies used were p-DNA-PKcs (p-S2056) ab18192 (Abcam), p-RET (Tyr 1062)-R sc-20252-R (Santa Cruz), pERK 1/2 (Thr 202/Tyr 204) sc-16982 (Santa Cruz), pAKT (phospho S473) ab66138 (Abcam). Images were taken using a Zeiss AxioImager Z1 microscope and an attached Zeiss AxioCam MRc5 camera. The level of staining was graded by a blinded pathologist and scaled from 0–3 (0—no staining, 1—light, 2-moderate, 3-high). Chi tests were performed to determine the significance of our findings. p values of ≤ 0.05 were considered significant.

## Results

**Fig 1:**
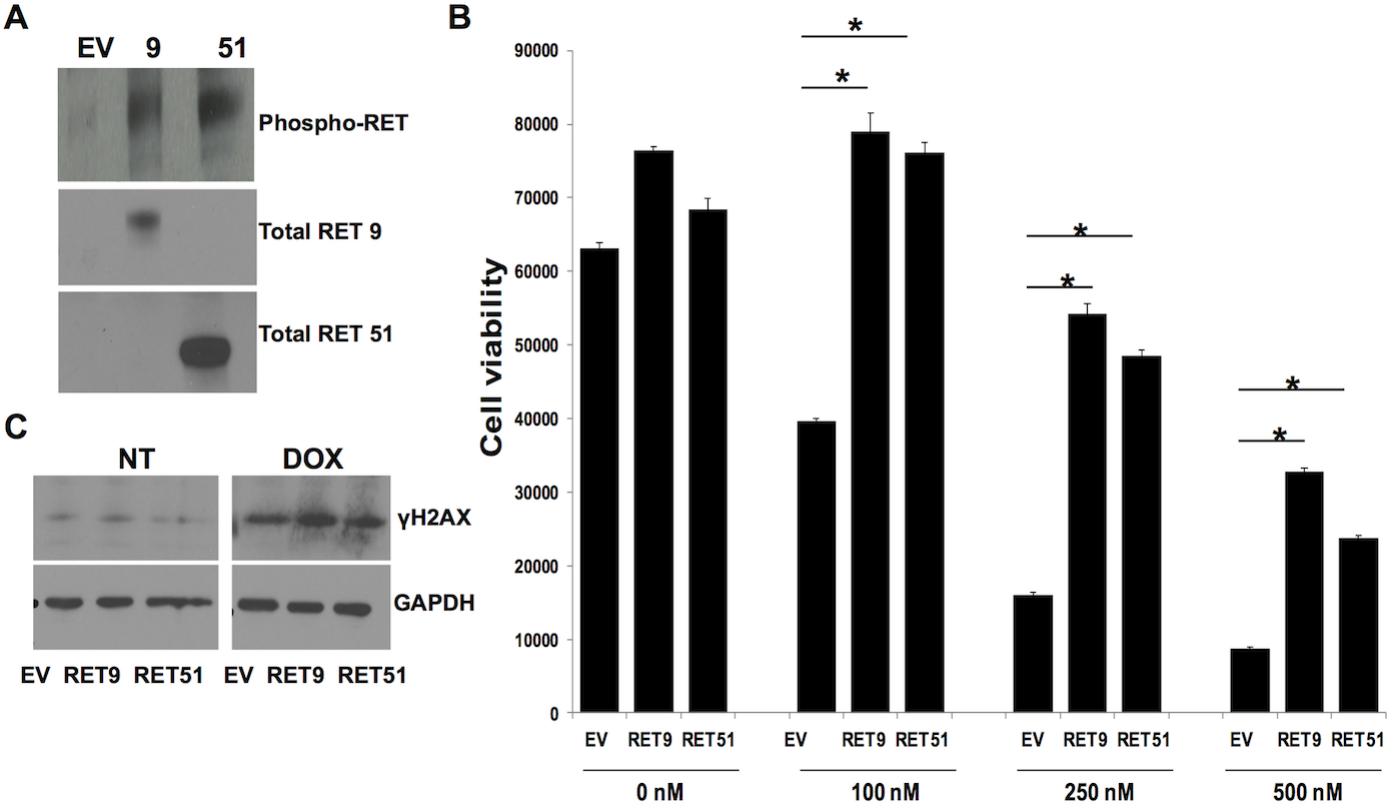
Increased expression of RET isoforms increases resistance to chemotherapy. A) Western blot analysis of RET lines show expression and phosphorylation of either RET 9 or RET 51 isoforms only in overexpressing lines and not in the control EV cells. B) RET expression causes cells to be resistant to doxorubicin treatment (DOX). *p≤ 0.01, error bars = s.d.

The RET signaling pathway has previously been analyzed in a modified SK-N-MC cell line that does not naturally express the RET receptor but does contain most of the known components of the RET signaling pathway [11–14]. This cell line was made to stably express a chimeric vector containing the EGF extracellular binding domain linked to the RET 9 isoform intracellular signaling domain or the RET 51 intracellular signaling domain. Published data show that when the RET signaling pathway is activated in these cells by binding of EGF present in the media, they become resistant to doxorubicin therapy [15]. Although a chimeric and artificial signaling molecule, this cell line, provided us with a cost effective option for identifying components of the RET pathway that are involved in chemoresistance. GDNF, the natural RET ligand, is not easily used in recombinant form nor is it easily added to the media in the quantities required for a proteomic screen. In Fig 1A it is apparent that in our standard growth conditions the cytoplasmic domain of both isoforms of RET are phosphorylated indicating activation of the pathway. In Fig 1B, all three cell lines were exposed to increasing concentrations of doxorubicin, a common chemotherapeutic agent utilized clinically and known to cause double stranded DNA breaks (DSB). Consistent with previous results [15], the presence of activated RET signaling reduces cell death despite identical cell growth and survival rates under non-genotoxic conditions (Fig 1B). These results suggest that RET signaling confers an increased capacitance to withstand DNA damage. We next sought to investigate differences in chromatin and associated proteins upon activation of RET signaling.

As an initial approach to investigate alterations in chromatin-templated activities in RET stimulated cells, we performed a proteomic screen on acid extractable proteins from nuclei isolated from both our control and RET activated cell lines. A simple protein extraction from cells using 0.4M sulfuric acid provides for extraction of histones as well as other basic nuclear associated proteins, e.g. HMG proteins, nucleosome binding proteins [16]. Proteins isolated from our nuclear preps were resolved by SDS-PAGE, visualized by Coomassie-staining, and subjected to in-gel trypsin digestion as previously reported in our laboratory [16–18]. Tryptic peptides were analyzed with high resolution tandem mass spectrometry (Thermo Velos Orbitrap mass spectrometer coupled to a Waters nanoACQUITY LC system) and identified with Mascot software as previously reported [8–10]. Since we were interested in chromatin-templated activities, we first looked for differences in levels of histone posttranslational modifications (PTMs) including acetylation and methylation. A quantitative analysis of histone PTMs in the three cell lines did not uncover bulk changes in PTM levels (data not shown). Of note, we were unable to identify phosphorylation sites by this method. We next analyzed the proteomic data for proteins, other than histones, that change nuclear levels in response to RET signaling. Using a quantitative analysis of the proteomic data [8–10], we identified a series of chromatin-associated proteins (486 proteins in total) with altered expression (108 of 486 showing altered expression) in RET stimulated cells—one of which was DNA-PKcs. The amount of DNA-PKcs isolated from nuclear associated protein preps was increased by ~24-fold in RET 9 and ~14-fold in RET 51 lines. Table 1 highlights proteins identified with a significant change in expression in both RET 9 and RET 51. A complete list is provided in S1 Table. Given the ability of RET signaling to promote chemoresistance, we were particularly interested in immediately investigating the effect DNA-PKcs was having in our model system of genotoxic stress.

**Table 1:** List of proteins found to be significantly altered in RET 9 and RET 51 lines compared to control lines.

Protein Name	RET 9	RET 51	Fold Change 9–51	Expression	Known Fxn
Nucleosome-binding protein1	9.954E-05	3.35E-06	13.07–16.29	Increased	Modifies chromatin structure for regulation of transcription and histone compaction
**Isoform 1 of DNA-dependent protein kinase catalytic subunit**	**2.396E-05**	**0.006**	**24.14–14.04**	**Increased**	**Binds chromatin and is required for NHEJ pathway of DNA damage**
Core histone macro-H2A2	0.007	0.006	6.63–6.71	Increased	Histone variant of H2A, represses transcription
Zinc finger protein 22	0.11	0.0003	6.4–5.22	Increased	Binds DNA to regulate transcription
U4/U6. U5 tri-snRNP-associated protein 1	0.02	0.02	8.24–6.58	Increased	Binds DNA and regulates RNA splicing
High mobility group protein	0.01	0.05	0.07–0.17	Decreased	Transcriptional regulator, chromatin condensation
Isoform 1 of Methyl-CpG-binding protein 3	0.001	0.001	0.18–0.18	Decreased	Recruits histone deacetylases and DNA methyltransferases
Isoform 2 of sister chromatid cohesion PDS5	0.001	0.001	0.23–0.23	Decreased	Involved in chromosome cohesion and DNA replication
Eukaryotic translation initiation factor 4E-binding protein 1	0.004	0.009	0.38–0.38	Decreased	Represses protein translation, involved in response to UV/IR
Isoform 1 of Nucleophosmin	0.0008	0.0006	0.49–0.53	Decreased	Histone assembly, cell proliferation

Fold change ≤1.5 is considered increased expression and between 0.2 and 0.6 is decreased expression.

The catalytic subunit of DNA-dependent protein kinase (DNA-PKcs) is a 460 kDa polypeptide member of the PI3k family that was initially discovered to be a key component in the double stranded DNA break repair pathway, non-homologous end-joining (NHEJ). The protein is ubiquitously expressed in cells and localizes to sites of DSB with the Ku70/80 heterodimer [17]. Upon recruitment to chromatin, DNA-PKcs becomes activated via autophosphorylation at serine 2056 to stabilize DNA binding and promote DSB repair via phosphorylation of H2AX. [17–19]. Previous work has demonstrated that mutation of the s2056 cluster significantly impairs NHEJ activity and increases radiosensitivity [20]. In addition, chemical inhibition of DNA-PKcs in breast and colon cancer cell lines increased chemosensitivity and reduced tumor growth *in vivo* [21]. Given the increased levels of DNA-PKcs associated with chromatin in our proteomic screen, we hypothesized that RET signaling promotes chemoresistance by inducing phosphorylation and activation of DNA-PKcs leading to a subsequent increase in the capacitance of cells to handle genotoxic stress. This hypothesis is further supported by data put forth by Rodemann and Chen labs and their work on the EGF receptor [22, 23].

### Phosphorylation of DNA-PKcs at s2056 is elevated in RET expressing cells and can be reduced by RET inhibition

**Fig 2:**
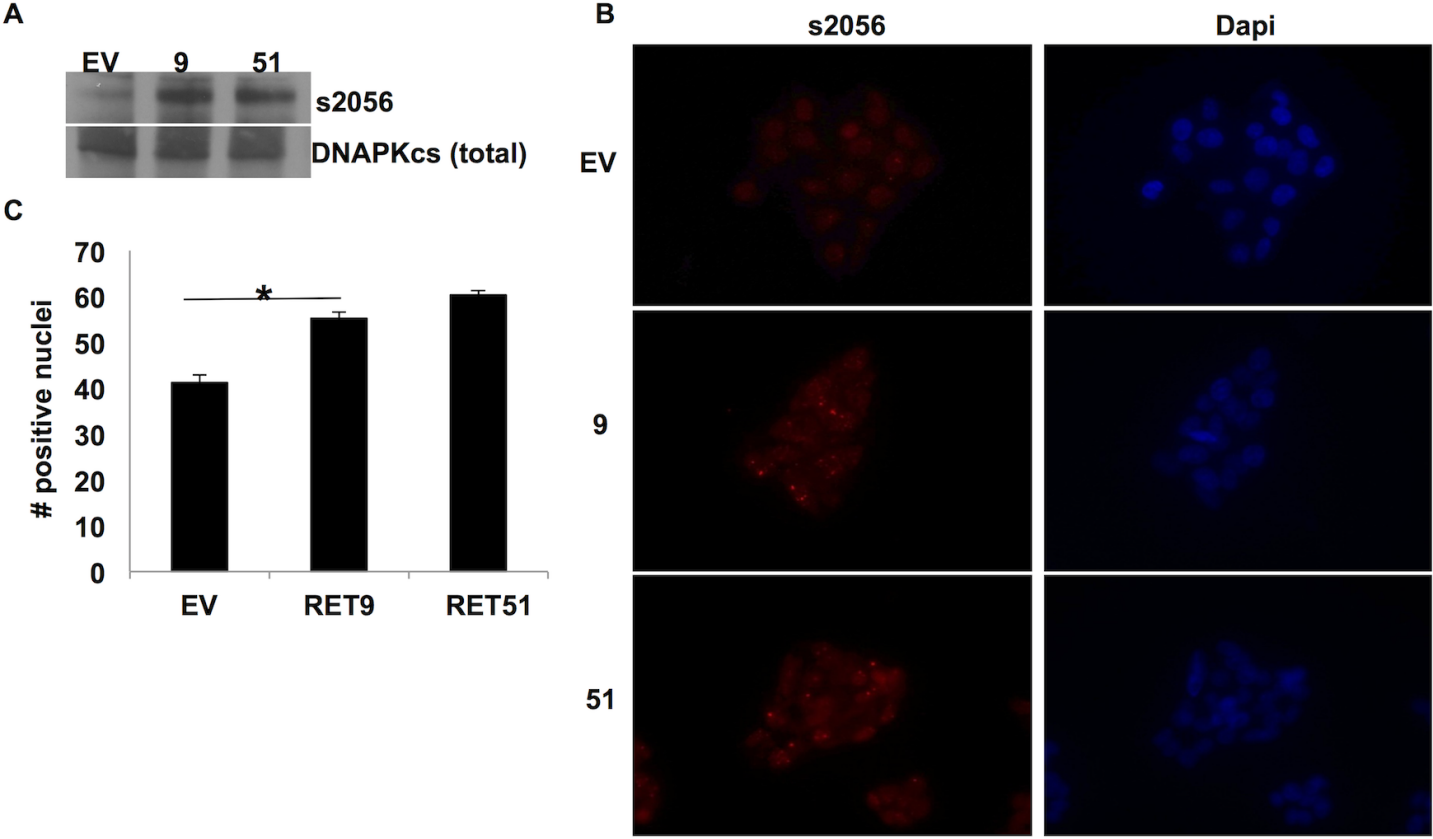
Phosphorylation of DNA-PKcs at s2056 is elevated in RET 9 and RET 51 cells. A) Western blot analysis shows phospho-s2056 (ps2056) (460 Kda) to be elevated in RET9 and RET 51 cell lysates. Total DNA-PKcs was used as loading control. B) Immunocytochemistry of cells plated in chamber slides and stained for ps2056 (red) and Dapi as counterstain (blue). C) ICC revealed a significant increase in ps2056 located in the nuclei of RET 9 and RET 51 cells compared to EV. * p ≤ 0.05, error bars = s.d.

To investigate this hypothesis we analyzed the level of total DNA-PKcs and s2056 phosphorylated DNA-PKcs in our three cells lines (EV, RET9, RET51) grown under normal growth conditions. As expected, there was no significant difference in the total levels of DNA-PKcs in whole cell lysate preps. Given that DNA-PKcs is a ubiquitously expressed protein we did not anticipate detecting changes in total levels, however upon probing for the activated form of DNA-PKcs phosphorylated at s2056 which is highly localized to the nucleus, we discovered that the levels were indeed increased in our RET expressing cell lines there-bye further confirming what we observed by mass spectrometry analysis (Fig 2A). Immunocytochemistry of this phosphorylated form of DNA-PKcs demonstrated that it was indeed present at increased levels in the nuclei of cells with activated RET signaling (Fig 2B and 2C).

**Fig 3:**
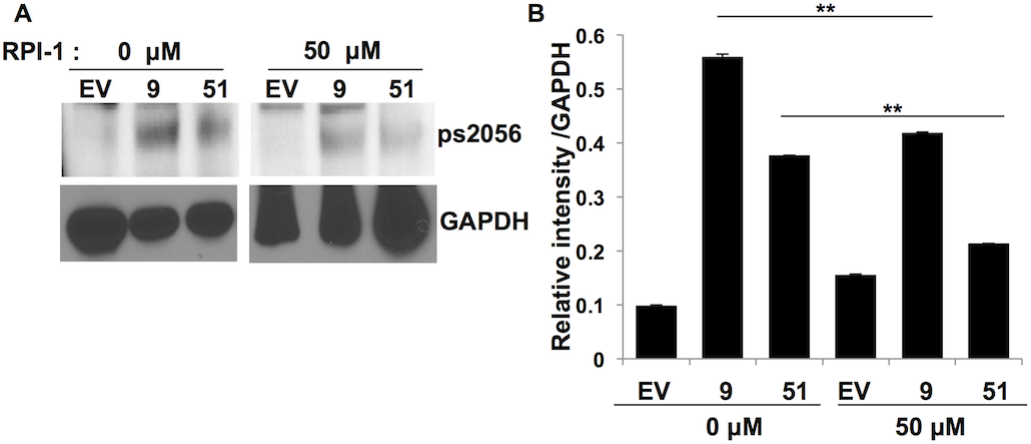
RET inhibition reduces phosphorylation of DNA-PKcs. EV, RET 9 and RET 51 cells were treated with the RET inhibitor RPI-1 for 24 hours before cells were harvested and analyzed by western blot. RPI-1 treatment significantly reduced phosphorylation of DNA-PKcs at site s2056 according to Image J analysis. GAPDH was used as loading control. **p ≤ 0.005, error bars = s.d.

After validating our mass spectrometric data we next questioned whether chemical inhibition of RET signaling would reduce phosphorylation of DNA-PKcs. Cells were grown in the presence or absence of RPI-1, a specific RET inhibitor [24]. In the absence of the chimeric oncogene (EV cells) or via chemical inhibition of RET activity we were able to significantly reduce phosphorylation of DNA-PKcs at s2056 further validating DNA-PKcs as a target of the RET signaling pathway (Fig 3).

### Inhibition of DNA-PKcs partially restores the chemosensitivity of RET expressing cells

**Fig 4:**
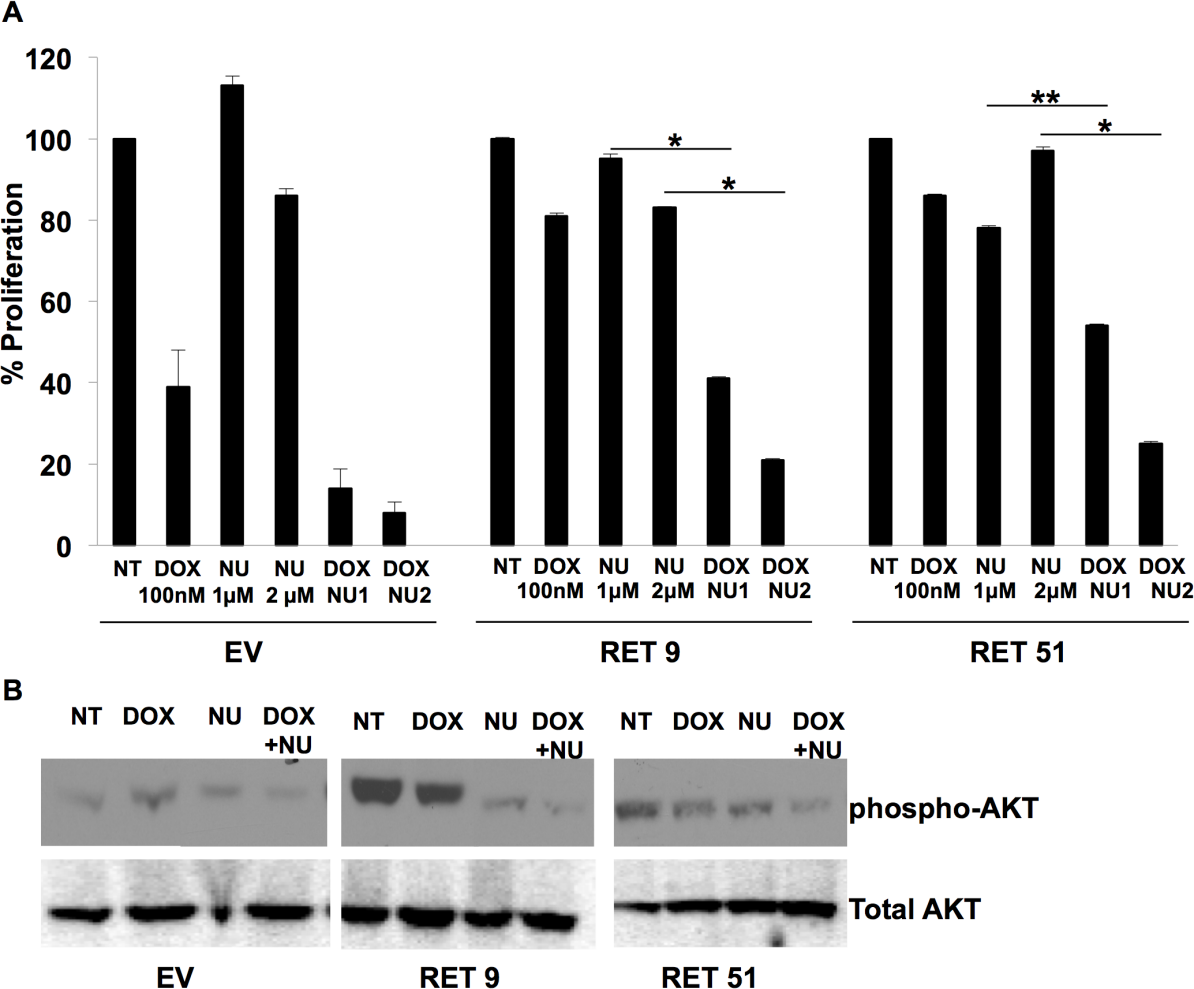
Inhibition of DNA-PKcs reduces chemoresistance in RET 9 and RET 51 cells. EV, RET 9 and RET 51 cells were treated with the DNA-PKcs inhibitor Nu7441 (NU) alone or with doxorubicin (DOX). EV proliferation was set at 100 and the % of EV for 9 and 51 were plotted. NU increased sensitivity to DOX in both the RET 9 and 51 cells. *p ≤ 0.05, **p ≤ 0.01, error bars = s.d. B) Western blot analysis of the DNA-PKcs target gene AKT showed decreased AKT phosphorylation with NU treatment indicating inhibition of DNA-PKcs activity.

Nu7441, a specific DNA-PKcs inhibitor developed from the wortmannin lead compound, has 100-fold specificity over other PI3 kinases and is effective in cell culture at low micromolar concentrations [25]. We hypothesized that treatment of these cells with this inhibitor should render cells with active RET signaling sensitive to chemotherapy. SK-N-MC cells grown in the presence of Nu7441 proliferated at similar rates to untreated cells as demonstrated in Fig 4A. Treatment with Nu7441 restored chemosensitivity to doxorubicin in the RET expressing cells. As expected due to the fact that this protein is critical for DNA damage response, this compound increased sensitivity of our control EV cell line to doxorubicin.

A primary target of DNA-PKcs enzymatic activity is AKT. Specifically it has been demonstrated that DNA-PKcs phosphorylates AKT at serine 473 [26]. This phosphorylation event is well documented as being important to cell survival [26]. Likewise, AKT is well known to be phosphorylated in response to RET activation at serine 473. Interestingly, inhibition of DNA-PKcs significantly reduced phosphorylation of AKT in our model system (Fig 4B) confirming effective inhibition of DNA-PKcs by Nu7441.

### Phosphorylation of DNA-PKcs at s2056 in human medullary thyroid cancer cells is affected by RET signaling and is important for chemosensitivity

**Fig 5:**
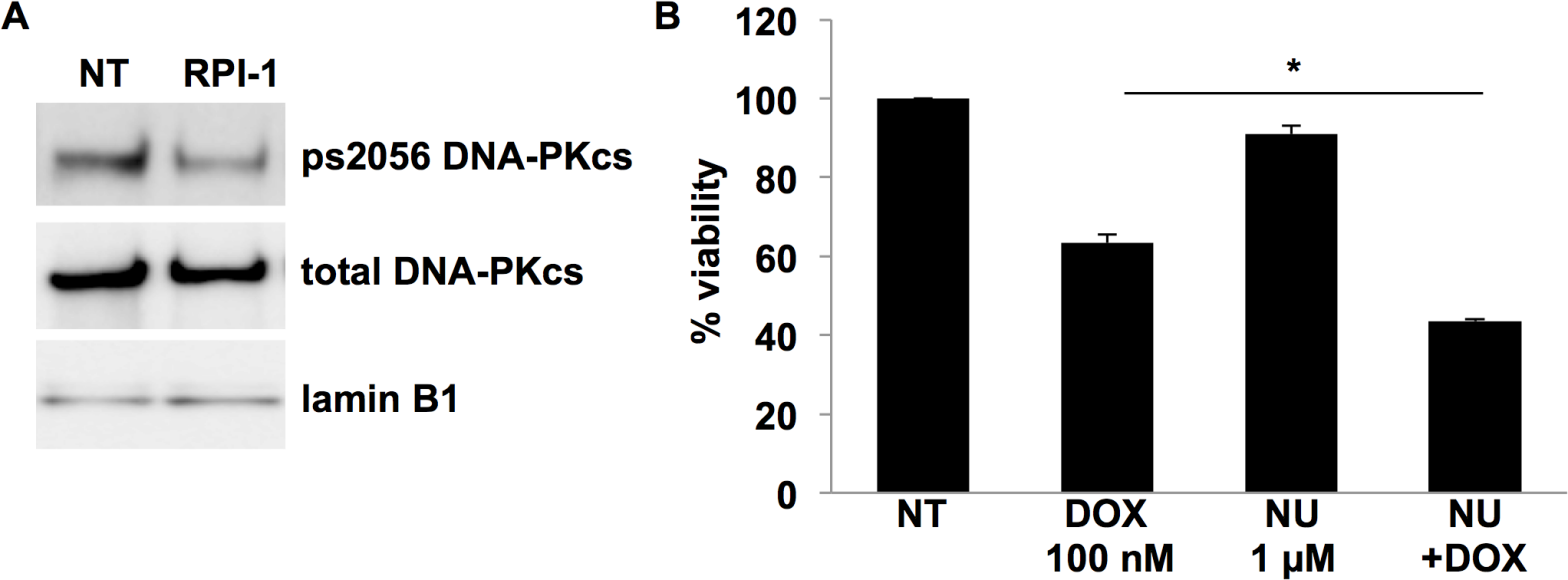
Inhibition of RET reduces phosphorylation of DNA-PKcs s2056 and blocking DNA-PKcs activity increases chemosensitivity. A) TT cells were treated with RPI-1 (5 μM) to inhibit RET for 24 hrs prior to nuclei isolation. Western blot analysis indicated that RET inhibition reduces DNA-PKcs s2056 phosphorylation. Total DNA-PKcs and Lamin B1 were used as loading controls. B) TT cells treated with Nu7441 (NU) (1 μM) to inhibit DNA-PKcs had a greater decrease in cell viability than DOX alone. *p ≤ 0.05, error bars = s.d.

In order to further confirm that DNA-PKcs is involved in the RET signaling pathway and not a phenomena unique to our screening system, our next set of experiments were moved into a more physiologically relevant system, MTC derived human TT cells. TT cells are ideal for studying components of the RET pathway because they harbor a MEN2A-like mutation in RET causing the pathway to be constitutively active [27]. We verify this by showing high levels of RET and pERK in TT cell lysates by western blot analysis (S1 Fig). Further analysis of nuclear extracts in human TT cells revealed as expected that DNA-PKcs is present, active, and phosphorylated at s2056 (Fig 5A). Importantly, the phosphorylation of DNA-PKcs at s2056 is blocked by inhibiting RET with the RPI-1 inhibitor (Fig 5A). Likewise, inhibition of DNA-PKcs with Nu7441 significantly increased TT cell sensitivity to doxorubicin indicated by a decrease in TT cell viability as compared to doxorubicin alone (Fig 5B). This data indicates that inhibition of DNA-PKcs may serve as an effective option for treatment of MTC and warrants further investigation.

### Phosphorylated DNA-PKcs is present in human medullary thyroid cancer

**Fig 6:**
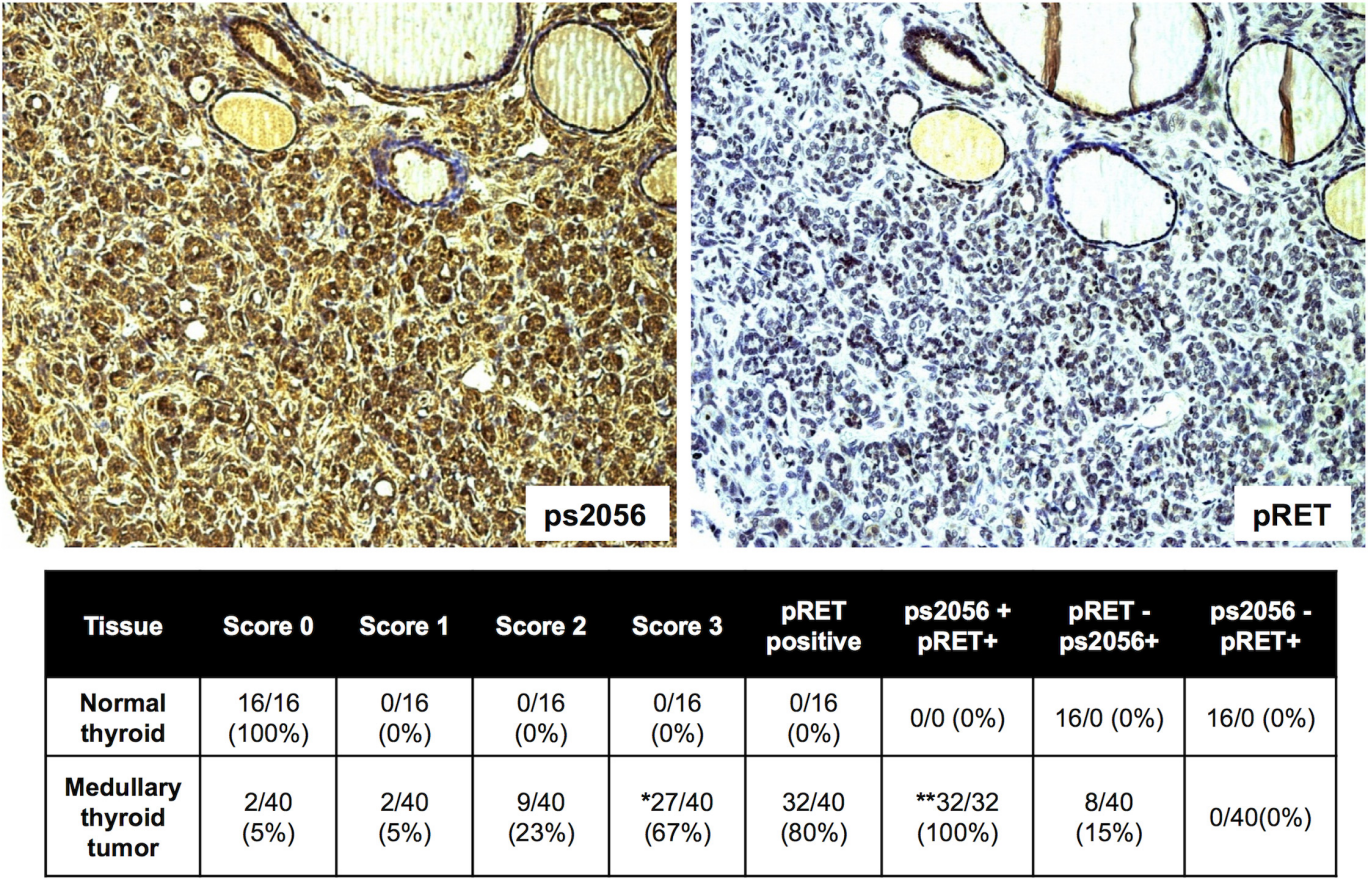
DNA-PKcs ps2056 is present in human medullary thyroid cancer and correlates with RET signaling. Immunohistochemistry analysis of tissue microarrays containing normal and MTC samples for phospho-s2056 (ps2056) and phospho-RET (pRET). Each tissue sample was given a score of 0 (no signal), 1 (weak), 2 (moderate), 3 (high) for ps2056 levels. Images represent a score of 3. *chi test p ≤ 0.01 for normal vs tumor ps2056, ** p ≤ 0.01 for normal vs. tumor +ps2056 +RET.

**Fig 7:**
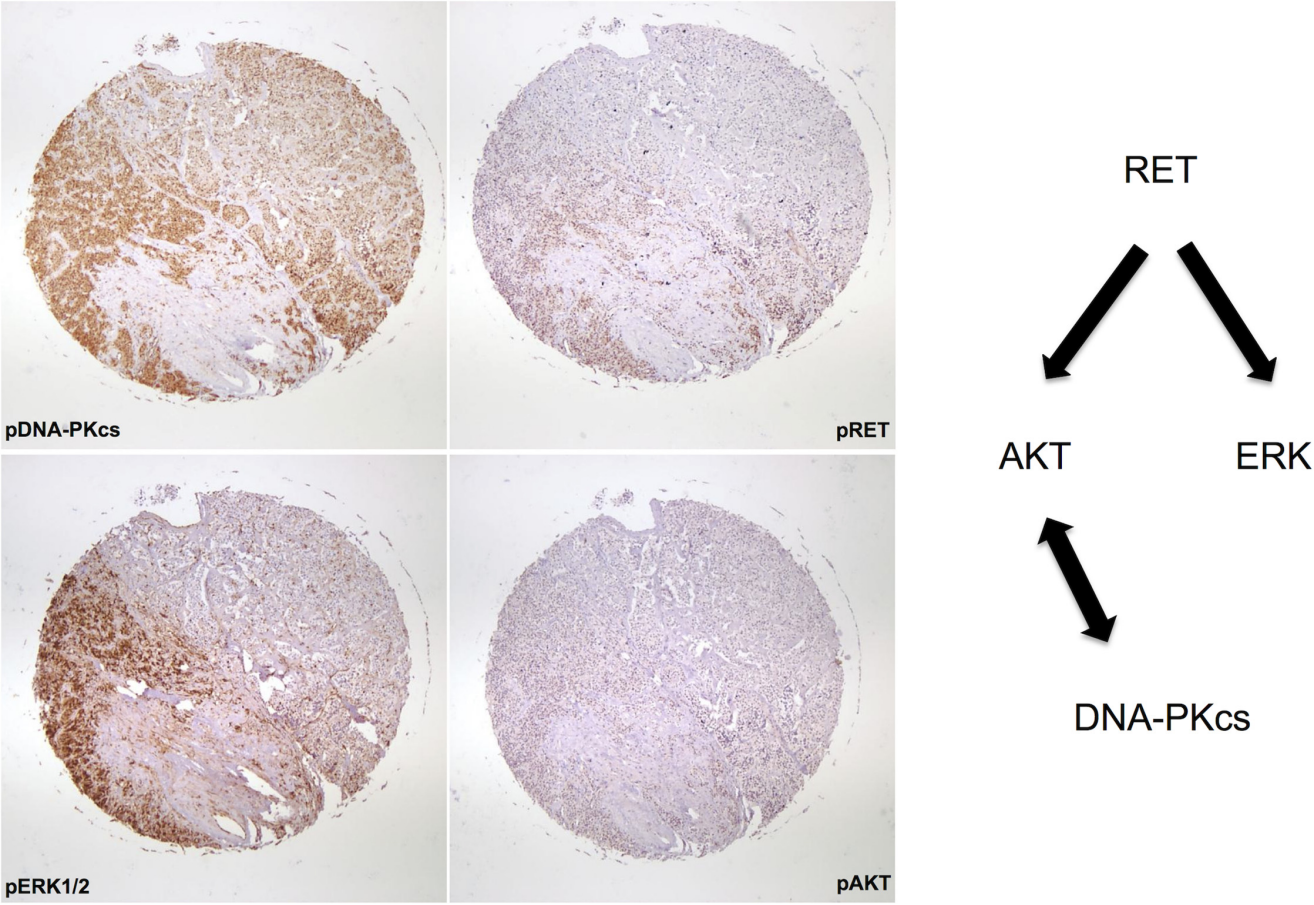
Components of an active RET signaling pathway are observed in MTC and correlate to DNA-PKcs. Immunohistochemistry of MTC array samples for phosphorylated ERK 1/2 and phosphorylated AKT indicated the presence of both of these components of an active RET signaling pathway in MTC similar to staining observed for ps2056 DNA-PKcs. 10x images of whole tissue array sample are shown.

With any potential drug target in the lab it is essential to verify at the outset that the target is present in human tumor samples. In order to validate DNA-PKcs as a potential target we obtained commercially available tissue microarrays of multiple MTC samples with normal thyroid controls. We were blinded to the genotype and patient outcomes of these samples. Of the MTC samples analyzed, 80% stained positive for phosphorylated RET, indicating activity of the RET signaling pathway (Fig 6). All samples that demonstrated RET activity also stained for phosphorylated DNA-PKcs at s2056. Notably, 0/16 control samples demonstrated expression of the phosphorylated form of DNA-PKcs. Fig 7 demonstrates staining of several components of the RET signaling pathway including phospho-ERK and phospho-AKT. This representative staining reveals a similar staining pattern for all four components including DNA-PKcs in these Stage II MTC tumor sample.

## Discussion

Despite a recent emphasis on the development of targeted therapy, the vast majority of cancer seen in the clinic is treated with genotoxic agents. As a result, chemoresistance remains the biggest challenge in cancer therapy. Identifying proteins that promote it is critical and could dramatically improve patient survival. Like most tumors with an activated RET proto-oncogene, medullary thyroid cancer is largely resistant to standard chemotherapy and radiation regimens. One hypothesis is that cells expressing the RET program are better equipped to process genotoxic stress. However, treatment of medullary thyroid cancer with RET inhibitors has shown modest efficacy to date in clinical trials for reasons not well understood [28]. It is believed that one of the main causes is simply that tumors are not homogenous and rely on multiple different proteins for survival. Although RET is a major player in the development of MTC, studies using targeted therapy have shown that it is not the only. This observation is therefore motivation to search for additional drug targets within oncogenic signaling pathways. In this study, we identify an unknown component of the RET signaling pathway which is critically involved in the repair of DNA damage, DNA-PKcs. Although the total amount of DNA-PKcs was not different in whole cell extracts of our control versus the RET expressing cell lines, the phosphorylated form of the protein which is highly associated with chromatin and active was significantly elevated. Activation of RET raised the levels of phosphorylated s2056 DNA-PKcs while inhibition of RET significantly reduced DNA-PKcs phosphorylation. Previous research has demonstrated that other receptor tyrosine kinase pathways are capable of activating DNA-PKcs, therefore; it is perhaps not surprising that our research has demonstrated this phenomena to occur in the RET signaling pathway in MTC as well [29]. This finding is significant because recent studies have highlighted the involvement of DNA-PKcs in chemoresistance and would therefore provide one mechanism by which RET and drives resistance [20–23]. Most importantly, our data shows that inhibition of DNA-PKcs restores chemosensitivity to our RET expressing cells. In order to demonstrate that activated DNA-PKcs is in fact a legitimate drug target we validated our results in human TT cells and probed tissue microarrays of medullary thyroid cancer for the presence of DNA-PKcs phosphorylated at s2056. We demonstrated the presence of the target in 95% of the tumor samples and 100% of those tumors with an active RET signaling pathway. These results suggest that DNA-PKcs would be an interesting drug target for medullary thyroid cancer and perhaps RET expressing tumors in general [30, 31]. By targeting multiple sites along the RET pathway, we may better be able to treat advanced MTC. Currently in the United States there are three phase I clinical trials testing DNA-PKcs inhibition in the treatment of advance solid tumors. (clinicaltrials.gov, 2014)

## Supporting Information

S1 Fig TT cells contain an active RET signaling pathway. (TIFF)

S1 Table Full List of proteins identified in screen to be altered in RET expressing cell lines. Fold change ≤1.5 is considered increased expression and between 0.2 and 0.6 is decreased expression.
(XLSX)
